# Amino acid metabolism genes associated with immunotherapy responses and clinical prognosis of colorectal cancer

**DOI:** 10.3389/fmolb.2022.955705

**Published:** 2022-08-05

**Authors:** Xinyi Peng, Ting Zheng, Yong Guo, Ying Zhu

**Affiliations:** ^1^ The First Clinical College of Zhejiang Chinese Medical University, Hangzhou, Zhejiang, China; ^2^ Department of Oncology, Zhejiang Provincial Hospital of Traditional Chinese Medicine, Hangzhou, Zhejiang, China; ^3^ Hangzhou Hikvision Digital Technology Co, Ltd, Zhejiang, China

**Keywords:** colorectal cancer, amino acid metabolism, immunity, risk signature, immunotherapy responses

## Abstract

Based on amino acid metabolism-related genes (AAMRGs), this study aimed at screening out key prognosis-related genes and finding the underlying correlation between the amino acid metabolism and tumor immune microenvironment of colorectal cancer. A total of 448 amino acid metabolism-related genes were obtained from MsigDB. The risk signature was built based on differential expression genes, univariate Cox, and LASSO analyses with 403 patients’ data downloaded from the TCGA database. Survival analysis and independence tests were performed to confirm the validity of the risk signature. Single-sample gene set enrichment analysis (ssGSEA), tumor mutation burden (TMB), the score of tumor immune dysfunction and exclusion (TIDE), the immunophenoscore obtained from The Cancer Immunome Atlas database, and the IC50 of drugs were used to find the relationship among the risk signature, immune status, immunotherapy response, and drug sensitivity of colorectal cancer. We identified five amino acid metabolism-related genes for the construction of the risk signature, including ENOPH1, ACAT1, ALDH4A1, FAS, and ASPG. The low-risk group was significantly associated with a better prognosis (*p* < 0.0001). In the entire set, the area under the curve (AUC) for 1, 3, and 5 years was 0.717, 0.734, and 0.764, respectively. We also discovered that the low-risk subgroup was related to more activity of immune cells, had higher expression of some immune checkpoints, and was more likely to benefit from immunotherapy. ssGSEA revealed that except the processes of glutamine histidine, lysine, tyrosine, and L-phenylalanine metabolism, the other amino acid metabolism pathways were more active in the samples with the low risk scores, whereas the activities of synthesis and transportation of most amino acids were similar. Hedgehog signaling, WNT/β-catenin signaling, mitotic, notch signaling, and TGF-β signaling were the top five pathways positively associated with the risk score. To sum up, AAMRGs were associated with the immune microenvironment of CRC patients and could be applied as biomarkers to predict the prognosis and immunotherapy response of patients.

## Introduction

According to the International Agency for Research on Cancer, colorectal cancer (CRC) is currently one of the most commonly diagnosed tumors and the leading causes of tumor death worldwide (Sung et al., 2021). In recent years, it has been confirmed that the immunotherapy which used immune checkpoint inhibitors (ICIs) in metastatic CRC with deficient mismatch repair (dMMR) status, significantly improved the 5-year survival rate and overall survival (OS) of patients (Buchler, 2022). However, the biomarkers that predict the efficacy of ICIs therapy, including programmed death protein legend 1(PD-L1), mismatch repair deficiency, and tumor mutation burden (TMB), are still unsatisfactory in identifying the whole beneficiaries (Jin et al., 2022). To overcome this dilemma, researchers are still searching for new predictive biomarkers. On the other side, while continuing to study the genes associated with the tumorigenesis and metastasis of CRC, researchers have also begun to try developing drugs that target metabolic dependencies of colorectal cancer.

It is an indisputable fact that cancer cells display different metabolic patterns compared with normal cells (Hanahan and Weinberg, 2011). In addition to glucose, tumor cells also differ in the uptake and secretion of several amino acids (Jain et al., 2012; Dunphy et al., 2018). Hosios et al. (2016) reported that it is amino acids rather than glucose that are the dominant source of cell mass in proliferating cancer cells. In addition, amino acids also provide plenty of nitrogen to generate hexosamines, nucleotides, and other nitrogenous compounds in rapidly proliferating cells (Choi and Coloff, 2019). The amino acid metabolism also influences tumor-specific immunity. Oh et al. (2020) found that inhibiting the glutamine metabolism not only inhibits tumor development but also suppresses the production and recruitment of myeloid-derived suppressor cells (MDSCs). In recent years, the combination of amino acids and nanotechnology has attracted attention in drug delivery strategy development, providing a new way for targeted delivery of anti-tumor drugs (Er et al., 2021). Therefore, with the continuous development of targeted cell metabolism therapy, amino acid metabolism also has become one of the focuses of attention. Strategies of targeting amino acid metabolism therapy include inhibiting amino acid transportation, blocking amino acid biosynthesis, and depleting amino acids (Butler et al., 2021).

Accumulating evidence has revealed that amino acid metabolism is involved in the development and proliferation of CRC, and targeting amino acid metabolism can be a promising anti-CRC therapy. For instance, arginine and glutamine are essential amino acids for colorectal cancer cells (Lieu et al., 2020). SLC6A14 is an arginine transporter, and it has been demonstrated that the upregulation of SLC6A14 plays a pathogenic role in CRC (Gupta et al., 2005). Najumudeen et al. (2021) found that SLC7A5, a glutamine antiporter, is a promising target for therapy-resistant KRAS-mutant CRC, as mutant KRAS changes the basal metabolism of the tumor, increasing glutamine consumption to enhance proliferation. Zhao et al. (2020) reported that the combination of glutaminase inhibitor CB-839 with capecitabine preferentially inhibits PIK3CA-mutant CRC.

These findings elucidated that amino acid metabolism plays a vital role in colorectal cancer and tumor-related immunity. However, current studies on amino acid metabolism are mostly limited to a single gene or single amino acid. The relationship between multi amino acid metabolism–related genes and CRC remains largely unknown. Thus, we applied bioinformatics techniques to investigate the role of amino acid metabolism–related genes (AAMRG) in the prognosis and tumor-related immunity of colorectal cancer.

## Methods

### Colorectal cancer data and amino acid metabolism gene collection

**FIGURE 1:**
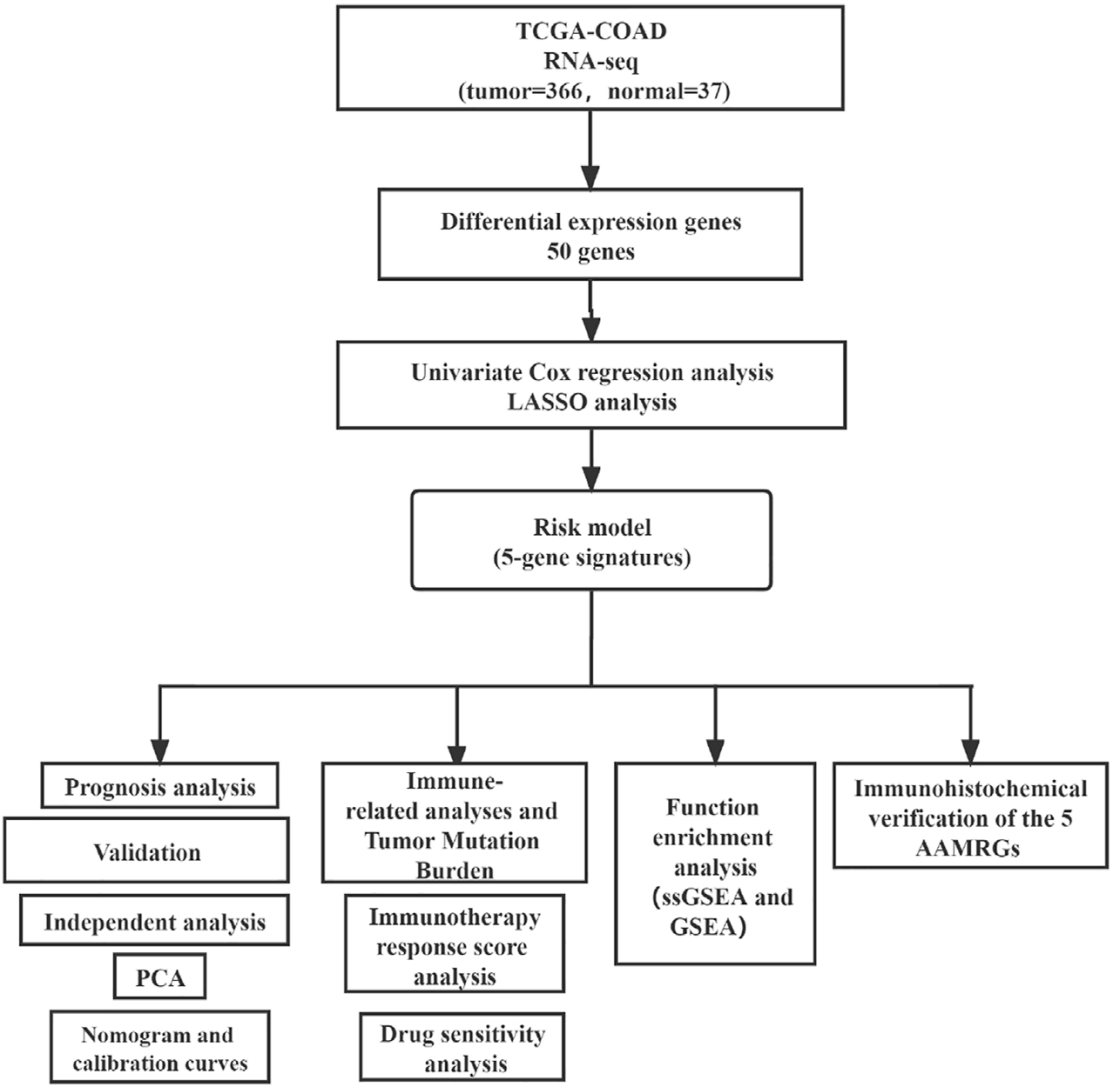
Flowchart for analyzing the amino acid metabolism–related genes of COAD.

The transcriptomic data (FPKM) of COAD with complete clinical data were obtained from The Cancer Genome Atlas (TCGA). This study only selected cases with primary colon cancer and clinical follow-up data for analysis. We removed genes with a mean expression less than 0.5, and FPKM data were transformed by log2 (FPKM + 1) for subsequent analysis. We screened out human amino acid metabolism pathways and corresponding 448 genes in the Molecular Signature Database (MsigDB) (Supplementary Tables S1, S2). The data analysis flowchart is shown in Figure 1.

### Identification of differential expression genes and construction of the risk signature

The limma package was used to identify the differential expression genes (DEGs), and the screening criteria were set as Log2FoldChange >1 and *p*-value < 0.05. The entire set was randomly separated into training and testing sets at a 1:1 ratio, using the “caret” package. Based on the result of DEGs, we implemented a univariate Cox analysis to select the genes significantly involved in prognosis risk. Thereafter, these genes were subjected to the least absolute shrinkage and selection operator (LASSO) analysis to exclude the over-fitting genes by the “glmnet” package.

### Validation of the AAMRG risk signature

We used the formula $risk\;score=\overset{n}{\underset{i=1}{\sum }}\left(Coef\left(RNA\;i\right)\times exp\left(RNA\;i\right)\right)$ to construct the AAMRG risk signature. Exp (RNA) is the expression value of the included AAMRGs, and coef (RNA) means the corresponding risk coefficient of each included gene. We selected the median value as the cutoff value to assign all samples into high-and low-risk subgroups.

Then, principal component analysis (PCA) was applied to evaluate the ability of the risk signature in distinguishing the high-risk samples from low-risk samples in the entire set. “Limma” and “scatterplot3d” packages were used in this process.

To investigate the prognostic prediction ability of genes, we performed survival analysis, the time-dependent receiver operating characteristic (timeROC), and calculated the area under the curve (AUC) based on the “survival,” “survminer,” and “timeROC” packages.

### Risk score and immune activity analysis

The immune score of each CRC sample was determined using the ESTIMATE method. Meanwhile, we used the single-sample Gene Set Enrichment Analysis (ssGSEA) method to evaluate the activity of immune infiltrating cells and compared the expression level of 31 immune checkpoints between the two groups. “Estimate,” “GSEABase,” and “GSVA” packages were used in this process.

Tumor mutation burden (TMB) files containing somatic mutation information were obtained from TCGA. The difference in TMB level between the two risk subgroups was evaluated and displayed by using “maftools,” “ggpubr,” and “ggplot2” packages. Kaplan–Meier (KM) analysis was used to analyze survival differences among subgroups with different TMB levels and risk statuses. “Survival” and “survminer” packages were utilized in this process.

### Risk score and immunotherapy response analysis

The tumor immune dysfunction and exclusion (TIDE) algorithm created by Liu et al. can predict the immunotherapy response of each patient (Jiang et al., 2018). Their research showed the outcome of TIDE was even more accurate than PD-L1 level and mutation load. The TIDE scores of samples were downloaded from the TIDE website (http://tide.dfci.harvard.edu).

We also downloaded the immunophenoscore data of samples from The Cancer Immunome Atlas (TCIA) database (https://tcia.at/home). TCIA provided this scoring scheme for quantifying immunophenotype, which could predict the responses of samples to anti-cytotoxic T lymphocyte antigen-4 (CTLA-4) and anti-programmed cell death protein 1 (anti-PD-1) therapies (Charoentong et al., 2017). The “ggplot2” and “ggpubr” packages were performed in the abovementioned processes.

The drug sensitivity data of 60 cell lines were acquired from the CellMiner website (https://discover.nci.nih.gov/cellminer/). We selected anti-CRC drugs commonly used in clinics for subsequent drug sensitivity analysis. The process of predicting the half-maximal inhibitory concentration (IC50) of these drugs was conducted using the “oncoPredict” packages (Maeser et al., 2021).

### Independence test of predictive ability and construction of the prognostic nomogram

We identified all the independent prognostic factors by univariate and multivariate Cox regression analysis with the “survival” package. Thereafter, we established a nomogram, simultaneously calculated the Concordance index (C-index) of this predictive model, and drew calibration curves to assess the OS probability at 3 and 5 years by applying the “rms” package.

### Functional enrichment analyses

We performed ssGSEA to evaluate the activity of amino acid metabolism–related pathway activity within the two risk subgroups, and a *p*-value < 0.05 was considered statistically significant. We also calculated the ssGSEA score of the hallmark pathway and found the pathways most correlated with risk score (*p*-value < 0.05). We displayed the top5 activated and suppressed pathways in the high-risk subgroup using the GSEA algorithm by clusterProfiler and enrichplot packages. The pathways of amino acid gene sets and hallmark were acquired from the MsigDB (http://www.gsea-msigdb.org/gsea/msigdb/collections.jsp) (Supplementary Table S3).

### Immunohistochemical verification of the identified AAMRG signature

The expression of AAMRGs in CRC tissues and normal tissues would be verified by the immunohistochemistry (IHC) and hematoxylin-eosin (HE) staining results from the Human Protein Atlas website (HPA, https://www.proteinatlas.org/).

### Statistical analysis

The statistical analyses were all implemented using R software (version 4.1.2). The quantitative variables and qualitative variables were shown in mean ± standard deviation and number (ratio%) format, separately. *t*-test or Wilcoxon test was used to compare the normal or non-normal distributed quantitative variables between the two subgroups. Chi-square analysis and Fisher’s test were used to compare the qualitative variables between the two subgroups.

## Results

### Construction of the risk signature on AAMRGs

**FIGURE 2:**
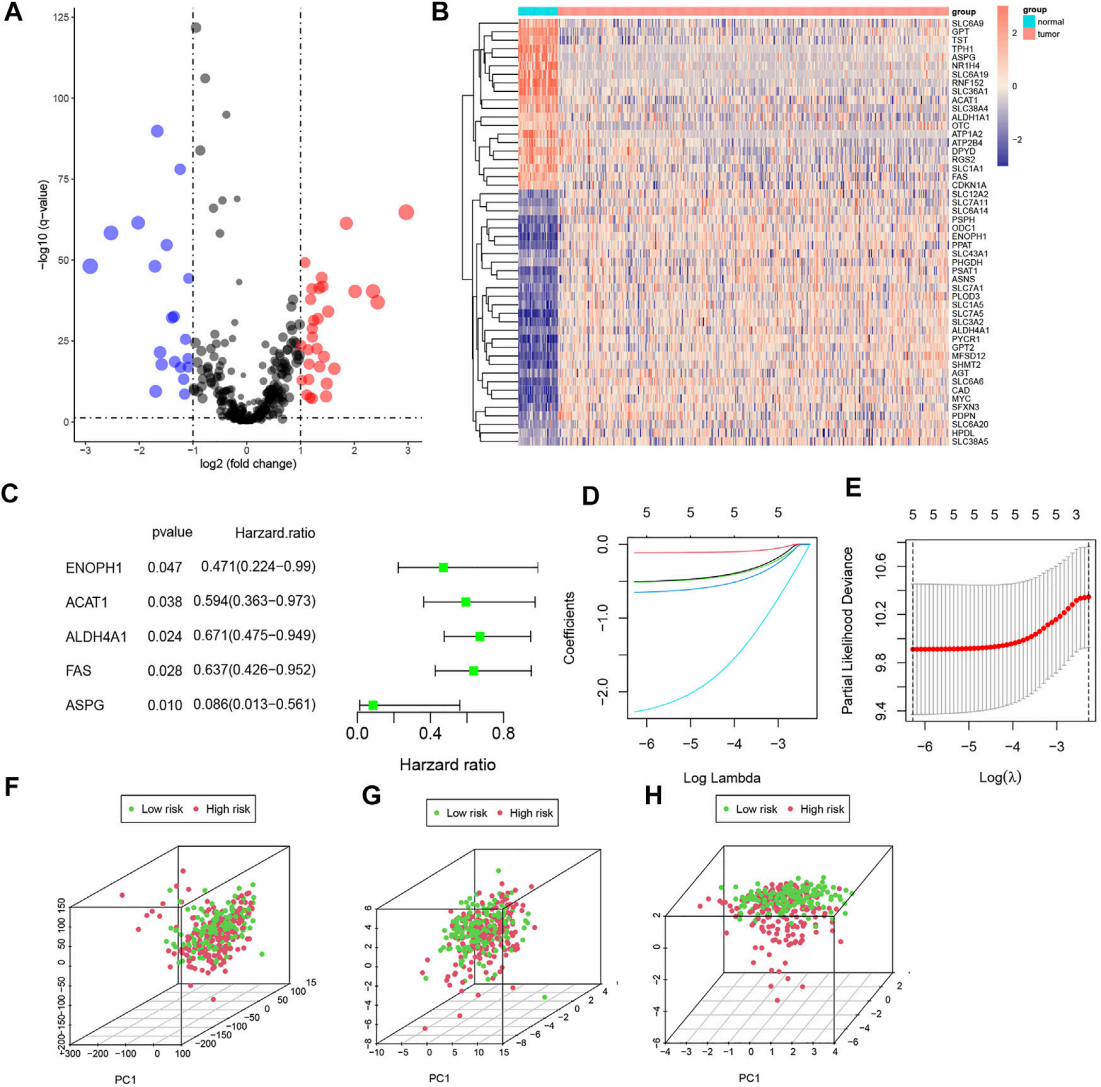
Identification of prognosis-related AAMRGs by differential expression gene (DEG) analysis, univariate Cox analysis, and LASSO analysis. **(A)** Volcano plot of AAMRGs (*p* < 0.05). **(B)** Heatmap of DEGs in colorectal cancer samples. **(C)** The forest plot of prognosis-related DEGs. **(D,E).** Construction of risk signature based on LASSO Cox analysis. **(F)** PCA of the entire gene set. **(G)** PCA of 50 DEGs set. **(H)** PCA of the five AAMRGs in the risk signature.

A total of 448 genes associated with amino acid metabolism were obtained from MsigDB, and 50 DEGs were identified (Figures 2A,B). Then, we randomly divided 366 COAD samples into the training set and test set, and the clinical characteristics of the two sets were shown in Table 1. In the training set, univariate Cox analysis screened out five genes related to the prognosis of CRC (*p* < 0.05) (Figure 2C). We further conducted a LASSO analysis to construct the risk signature (Table 2). The risk score was determined as follows, risk score = −0.5068×ENOPH1 + −0.1147×ACAT1 + −0.5140×ALDH4A1 + −0.6532×FAS + −2.2738×ASPG. All five genes were protective factors. Depending on the median value, we assigned samples to high-risk and low-risk subgroups (Figures 2D and E). The results of PCA proved that five AAMRGs in the risk signature had elevated efficiency and could separate the low- and high-risk groups well (Figures 2F–H).

**TABLE 1:** Characteristics of patients in the training and testing sets.

	Total (*n* = 366)	Training set (*n* = 184)	Validation set (*n* = 182)	*p*-value
Age				
<65	147 (40.2%)	72 (39.1%)	75 (41.2%)	>0.05
≥65	219 (59.8%)	112 (60.9%)	107 (58.8%)	
Gender				
Female	166 (45.4%)	81 (44.0%)	85 (46.7%)	>0.05
Male	200 (54.6%)	103 (56.0%)	97 (53.3%)	
T stage				
T1	11 (3.0%)	8 (4.3%)	3 (1.6%)	>0.05
T2	66 (18.0%)	35 (19.0%)	31 (17.0%)	
T3	251 (68.6%)	120 (65.2%)	131 (72.0%)	
T4	38 (10.4%)	21 (11.4%)	17 (9.3%)	
N stage				
N0	217 (59.3%)	114 (62.0%)	103 (56.6%)	>0.05
N1	85 (23.2%)	38 (20.7%)	47 (25.8%)	
N2	64 (17.5%)	32 (17.4%)	32 (17.6%)	
M stage				
M0	307 (83.9%)	150 (81.5%)	157 (86.3%)	>0.05
M1	53 (14.5%)	30 (16.3%)	23 (12.6%)	
Unknow	6 (1.6%)	4 (2.2%)	2 (1.1%)	
Pathologic stage				
Stage Ⅰ	63 (17.2%)	37 (20.1%)	26 (14.3%)	>0.05
Stage Ⅱ	140 (38.3%)	66 (35.9%)	74 (40.7%)	
stage Ⅲ	99 (27.0%)	43 (23.4%)	56 (30.8%)	
stage Ⅵ	53 (14.5%)	30 (16.3%)	23 (12.6%)	
Unknow	11 (3.0%)	8 (4.3%)	3 (1.6%)	

**TABLE 2:** Prognostic genes generated by LASSO Cox analysis.

Gene	Full name	Coef
ENOPH1	Enolase-phosphatase 1	−0.506778617
ACAT1	Acetyl-CoA acetyltransferase 1	−0.114712865
ALDH4A1	Aldehyde dehydrogenase 4A1	−0.513999531
FAS	Fas	−0.653248882
ASPG	Asparaginase	−2.273842625

### Survival analysis and validation of the risk signature

**FIGURE 3:**
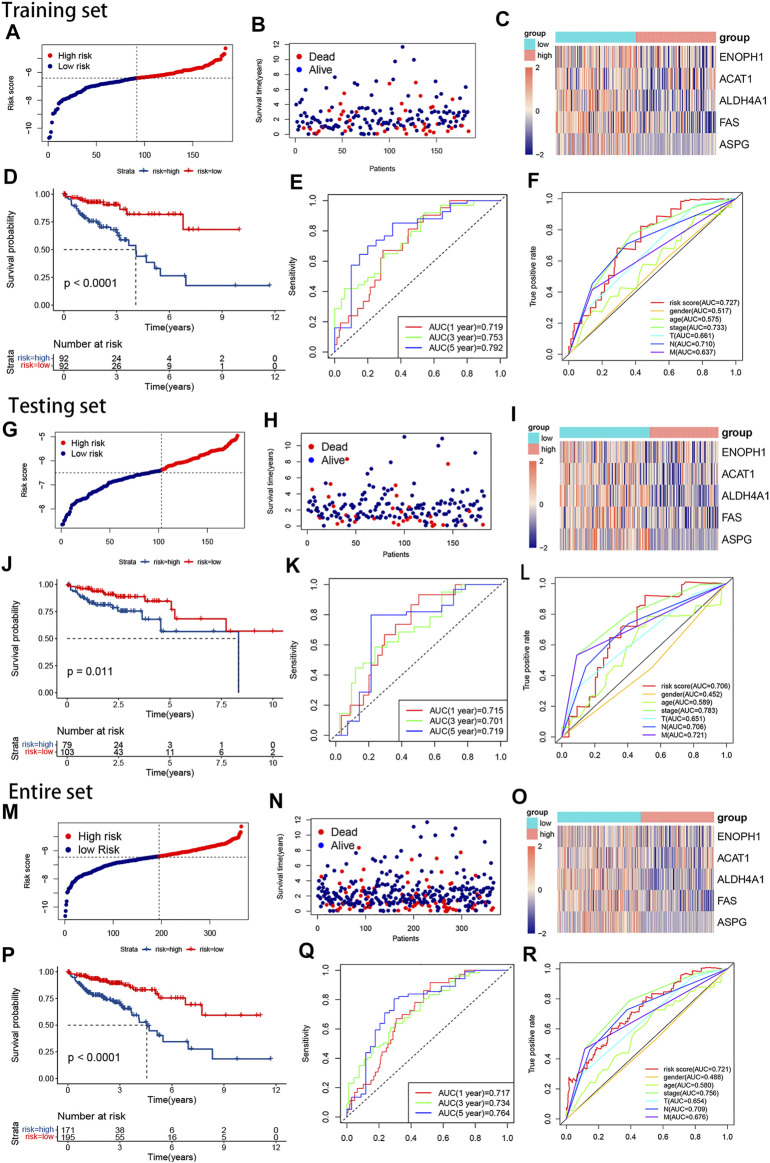
The plots of survival analyses, the distribution of patients’ survival times and survival status, and the heatmap of the expression of the five AAMRGs in the training set, the testing set, and the entire set, respectively. **(A,G,M)** The plots of risk score in the training set, the testing set, and the entire set, respectively. **(B,H,N)** The distributions of patients’ survival times and survival status in the training set, the testing set, and the entire set, respectively. **(C,I,O)**. Heatmaps of the expression matrix of the five AAMRGs in the training set, the testing set, and the entire set, respectively. **(D,J,P)** KM curves in the training set, the testing set, and the entire set, respectively. **(E,K,Q)** Time-dependent ROC curves in the training set, the testing set, and the entire set, respectively. **(F,L,R)** Clinical feature ROC curves in the training set, the testing set, and the entire set, respectively.

Our risk signature split the samples into high-risk and low-risk subgroups (Figures 3A, B, G, H, M, and N), and the heatmaps reflected the 5 AAMRGs were downregulated in the high-risk groups (Figures 3C, I, and O). KM analysis showed that the high-risk subgroup had a significantly worse prognosis than the low-risk subgroup (*p* < 0.0001) (Figure 3D). This outcome could be observed in the testing set and the overall set as well (Figures 3J and P). The accuracy of the risk signature was investigated by calculating the AUCs for 1, 3, and 5 years. In the training set, the AUCs for 1, 3, and 5 years were 0.719, 0.753, and 0.792, respectively (Figure 3E). In the testing set, the AUCs for 1, 3, and 5 years were 0.715, 0.701, and 0.719, respectively (Figures 3K). In the entire set, the AUCs for 1, 3, and 5 years were 0.717, 0.734, and 0.764, respectively (Figure 3Q). The ROC curves for clinical characteristics of each set are displayed in Figures 3F, L, and R. These outcomes illustrated that our risk signature was stable and did well in predicting the overall survival of CRC.

### Tumor mutation burden and immune-related analyses

**FIGURE 4:**
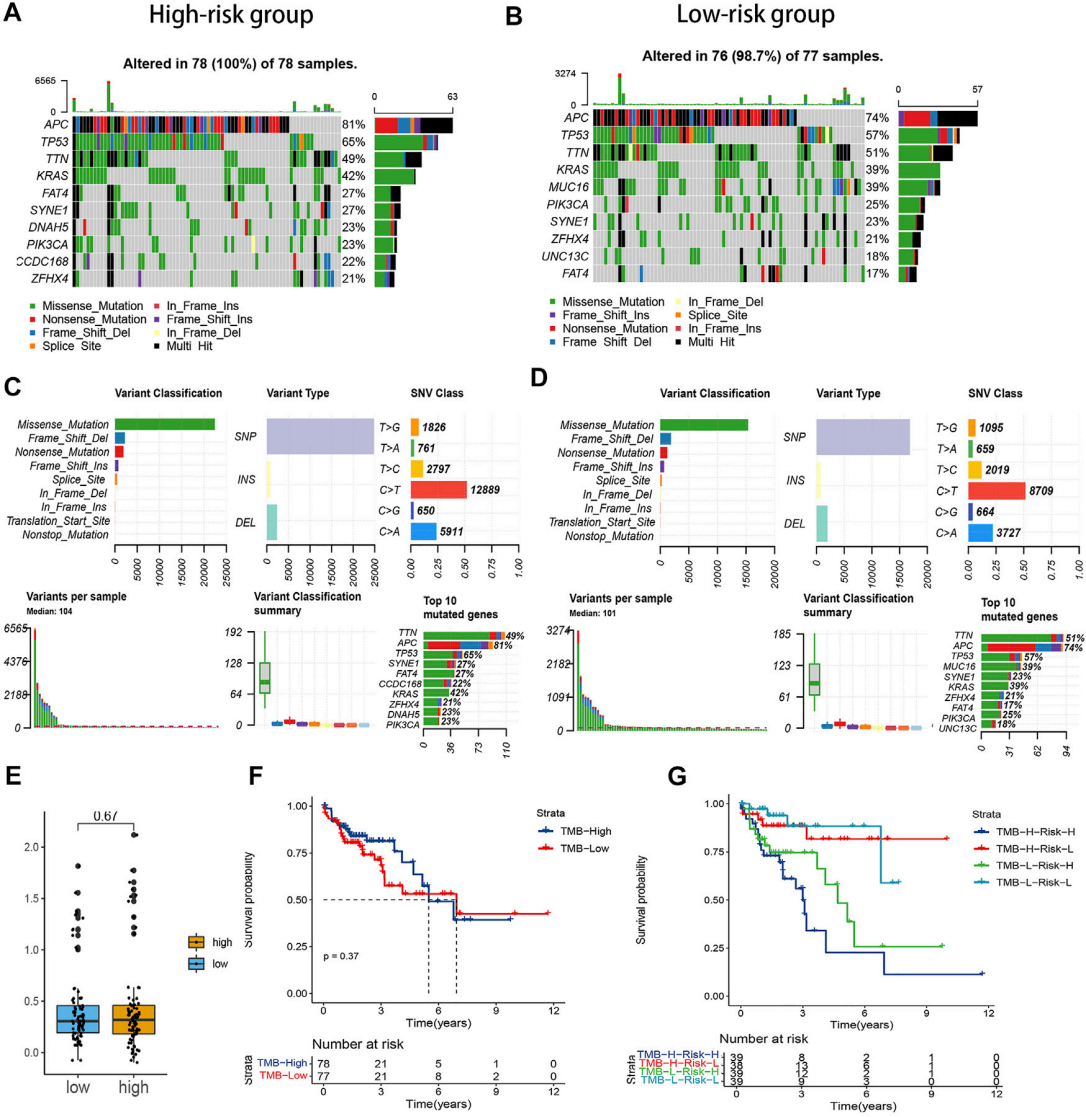
Correlation between the risk signature and tumor mutation burden (TMB) level. **(A,B).** Waterfall plot of the top ten genes’ TMB status in the two groups. **(C,D).** Summary of the maf files of the two groups. **(E)** Boxplot of the comparison of TMB levels in the two groups. **(F)** Survival analysis of the patients with different TMB levels. **(G)** Survival analysis of the patients with different TMB and risk levels.

As shown in Figures 4A–D, the TMB was a little higher in the high-risk subgroup, and the genes in the top ten mutation rates also differed between the two groups. Nevertheless, the TMB had no significant difference in the two subgroups on the whole (Figure 4E). To explore whether the risk signature or TMB is better in predicting survival, we separate the samples into high- and low-mutation subgroups based on the TMB value. There was no statistical difference between the two groups (Figure 4F). Whereas the low-mutation-low-risk group had the highest survival rate, and the high-mutation-high risk had the lowest survival rate (Figure 4G). This suggested that, compared with TMB, our model’s prediction ability was stronger.

**FIGURE 5:**
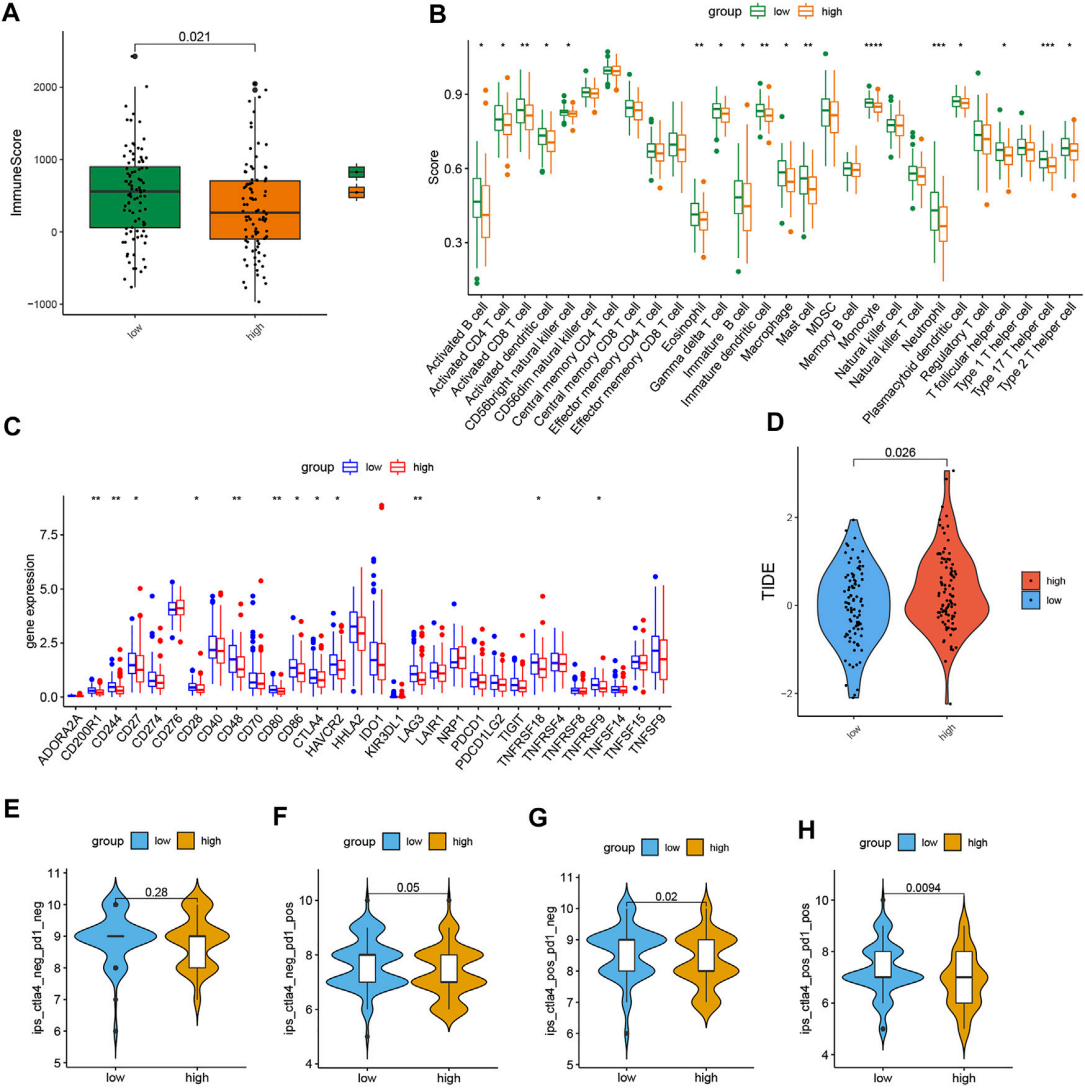
Comparison of immune-related analyses and immunotherapy response in the two subgroups. **(A)** Boxplot of the ESTIMATE score in the two subgroups. **(B)** Boxplot of the activity of immune infiltration cells in the two groups. **(C)** Boxplot of the expression level of immune checkpoint in the two subgroups. **(D)** TIDE score. **(E–H)** Immunophenoscore difference of COAD with different status of CTLA or PD-1. (*p* < 0.05 *, *p* < 0.01 **, *p* < 0.001 ***, and *p* < 0.0001 ****).

The outcome of ESTIMATE revealed that the high-risk subgroup had a lower immune score (Figure 5A). Next, as shown in the comparison plot of each immune cell activity, the activity of the CD4^+^ T cell, activated CD8^+^ T cell, T follicular helper (Tfh) cell, type 17 helper cell (Th17), type 2T helper cell (Th2), activated/immature/plasmacytoid dendritic cell, activated/immature B cell, macrophage, MDSC, monocyte, neutrophil, eosinophil cell, and mast cell decreased in the high-risk group (Figure 5B). The expression of plenty of immune checkpoints in the high-risk group was also reduced, including CD200R1, CD244, CD27, CD28, CD48, CD80, CD86, CTLA4, HAVCR2, LAG3, HHLA2, TNFRSF18, and TNFRSF9 (Figure 5C). Particularly, the expression of CTLA-4 and LAG3 was lower in the high-risk group. In addition, though PDCD-1 (PD-1) and CD274 (PD-L1) did not differ significantly between the two groups, there was still a downward trend in the high-risk group. The outcomes of the above immune-related analysis suggested that the risk signature was correlated to the immune landscape of CRC.

### Immunotherapy response analysis

We also found that the TIDE score in the low-risk group was strongly lower, which means low-risk patients may respond better to immunotherapy (Figure 5D). Furthermore, the plot of immunophenoscore from the TCIA database showed that low-risk patients with double-positive CTLA4 and PD-1 and single-positive CTLA4 or PD-1 had higher immunophenoscores, which means low-risk patients might benefit more from anti-PD and anti-CTLA4 therapies (Figures 5E–H). These results proved that the risk signature has the potential to be applicated to predict the immunotherapy response of CRC patients.

### Drug sensitivity prediction

**FIGURE 6:**
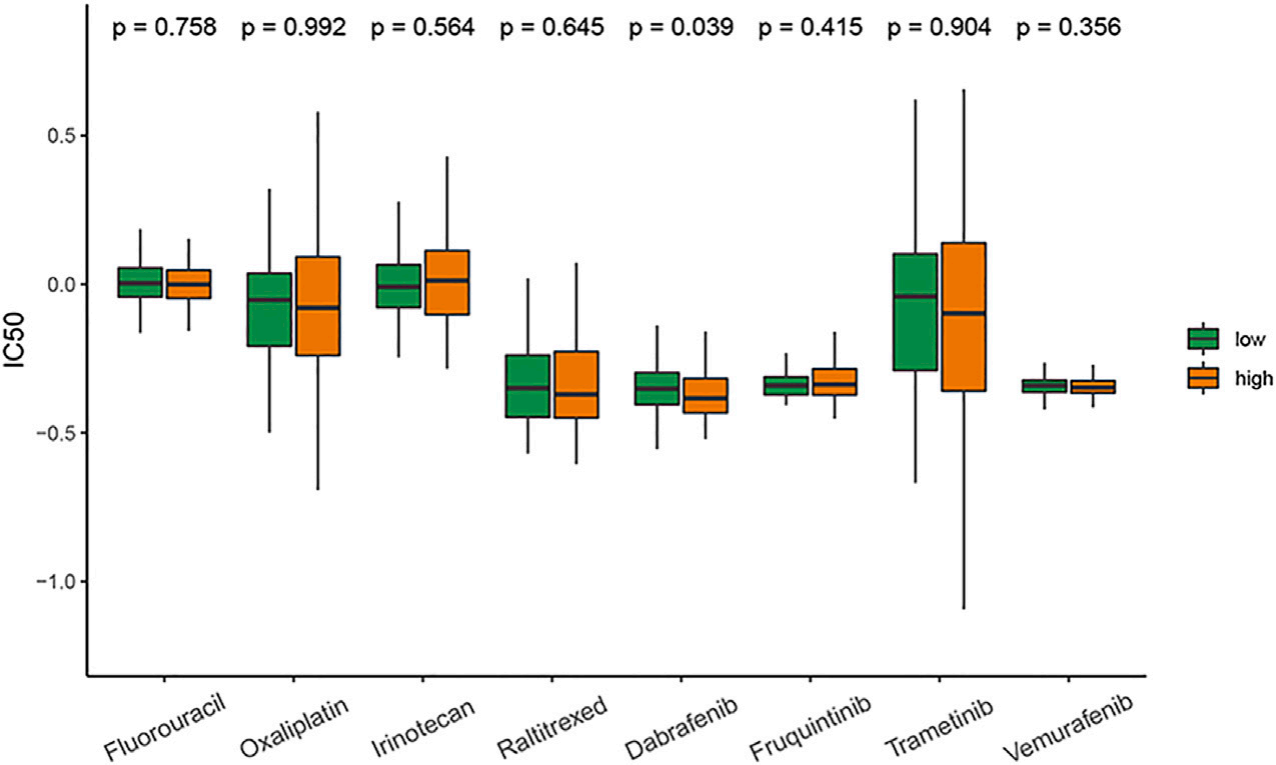
Comparison of drug sensitivity between the high- and low-risk groups.

The results showed that except for dabrafenib, the sensitivity to commonly used antitumor drugs for CRC in the two groups was similar. The patients with high-risk scores had higher sensitivity to dabrafenib (Figure 6).

### Independent prognostic analysis and construction of the prognostic nomogram

**FIGURE 7:**
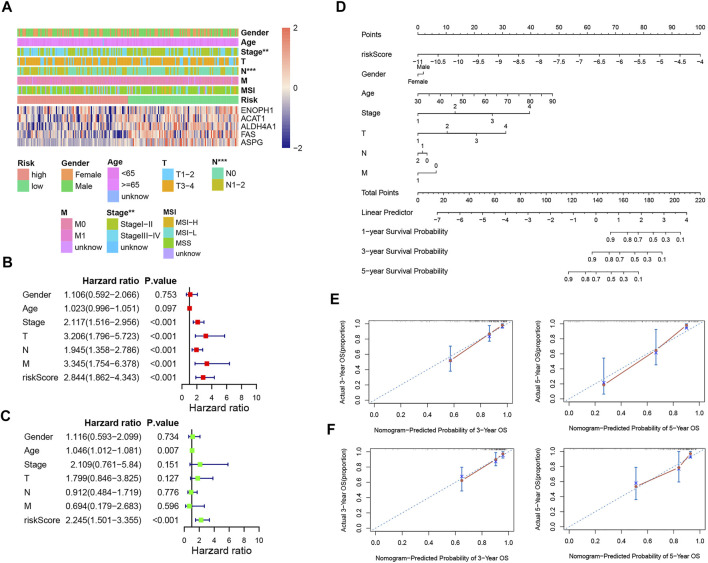
Independent prognostic analysis and construction of the prognostic nomogram. **(A)** Correlation between the risk signature and the clinical factors. **(B,C)** Forest plots for univariate COX and multivariate COX analyses based on risk score and the clinical factors. **(D)** Nomogram of COAD patients. **(E)** Calibration curves for the prediction of 3- and 5-year overall survival of COAD patients in the training set. **(F)** Calibration curves for the prediction of 3- and 5-year overall survival of COAD patients in the training set.

We employed chi-analysis to assess the association of the risk signature with clinical characteristics of CRC. The result indicated that the patients with high-risk scores may present more lymph node metastasis and more advanced stage (Figure 7A). Combined with clinical factors, the outcomes of univariate Cox analysis exhibited that risk score, T, N, M, and stage were independent predictive factors (Figure 7B). In addition, multivariate Cox analysis suggested age and risk score were independent predictive factors (Figure 7C). We also built a nomogram and drew the calibration curve plot of 3- and 5-year OS probability (Figure 7D). The C-indexes of the training set and testing set nomogram model were 0.809 and 0.808, respectively. As shown in the figure, the 3- and 5-year survival time predictive values for the two subgroups were both similar to the corresponding real survival time. These results indicated the excellent prediction ability of this nomogram (Figures 7E and F).

### Enrichment analysis of amino acid metabolism pathways and hallmark pathways

**FIGURE 8:**
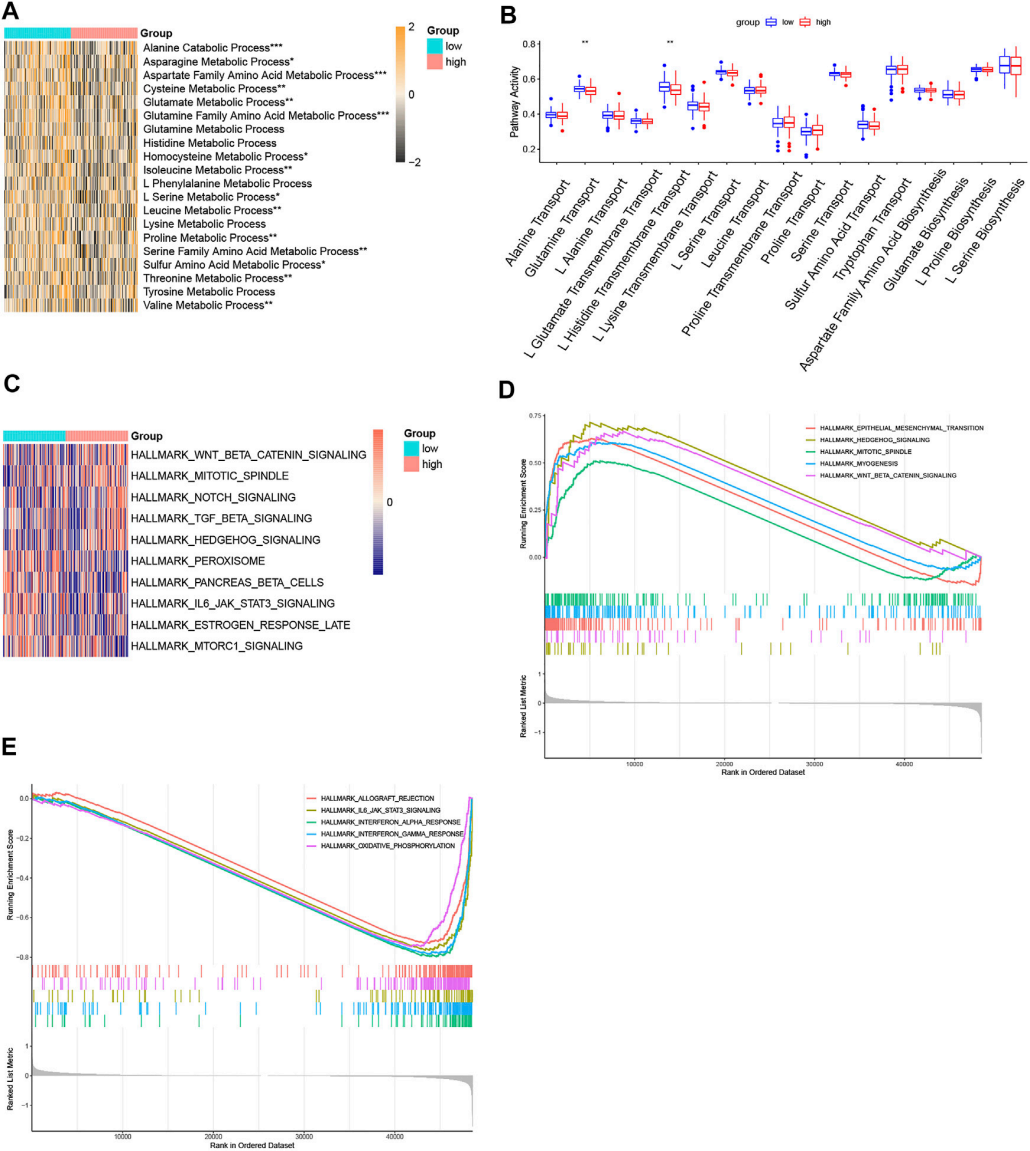
Function enrichment analyses. **(A)** Heatmap of ssGSEA of the amino acid metabolism pathways. **(B)** Boxplot of ssGSEA of the amino acid synthetic and transport pathways. **(C)** Heatmap of the top five positive or negative related with risk score. **(D)** GSEA plot of the top five activated pathways with the highest enrichment scores. **(E)** GSEA plot of the top five suppressed pathways with the lowest enrichment scores. (*p* < 0.05 *, *p* < 0.01 **, *p* < 0.001 ***, and *p* < 0.0001 ****).

The comparison of amino acid pathway activity in the two risk subgroups was visualized in heatmaps based on ssGSEA analysis. Except for the processes of glutamine histidine, lysine, tyrosine, and L-phenylalanine metabolism, the other amino acid metabolism pathways were more active in the patients with low-risk scores (Figure 8A). Except for the processes of glutamine transport and L-histidine transmembrane transport, the synthesis and transportation of most amino acids were similar in the two risk subgroups (Figure 8B). The hallmark pathways most associated with risk scores were visualized in the heatmap (Figure 8C). Hedgehog signaling, WNT/β-catenin signaling, mitotic, notch signaling, and TGF-β signaling were the top five pathways positively associated with the risk score. Peroxisome, pancreas beta cells, IL6/JAK/STAT3 signaling, estrogen response late, and mTORC1 signaling were the top five pathways negatively associated with risk score. In addition, the GSEA plots showed the top five enrichment scores of pathways that were activated or suppressed in the high-risk group (Figures 8D and E).

### The verification of the five AAMRGs in the Human Protein Atlas

**FIGURE 9:**
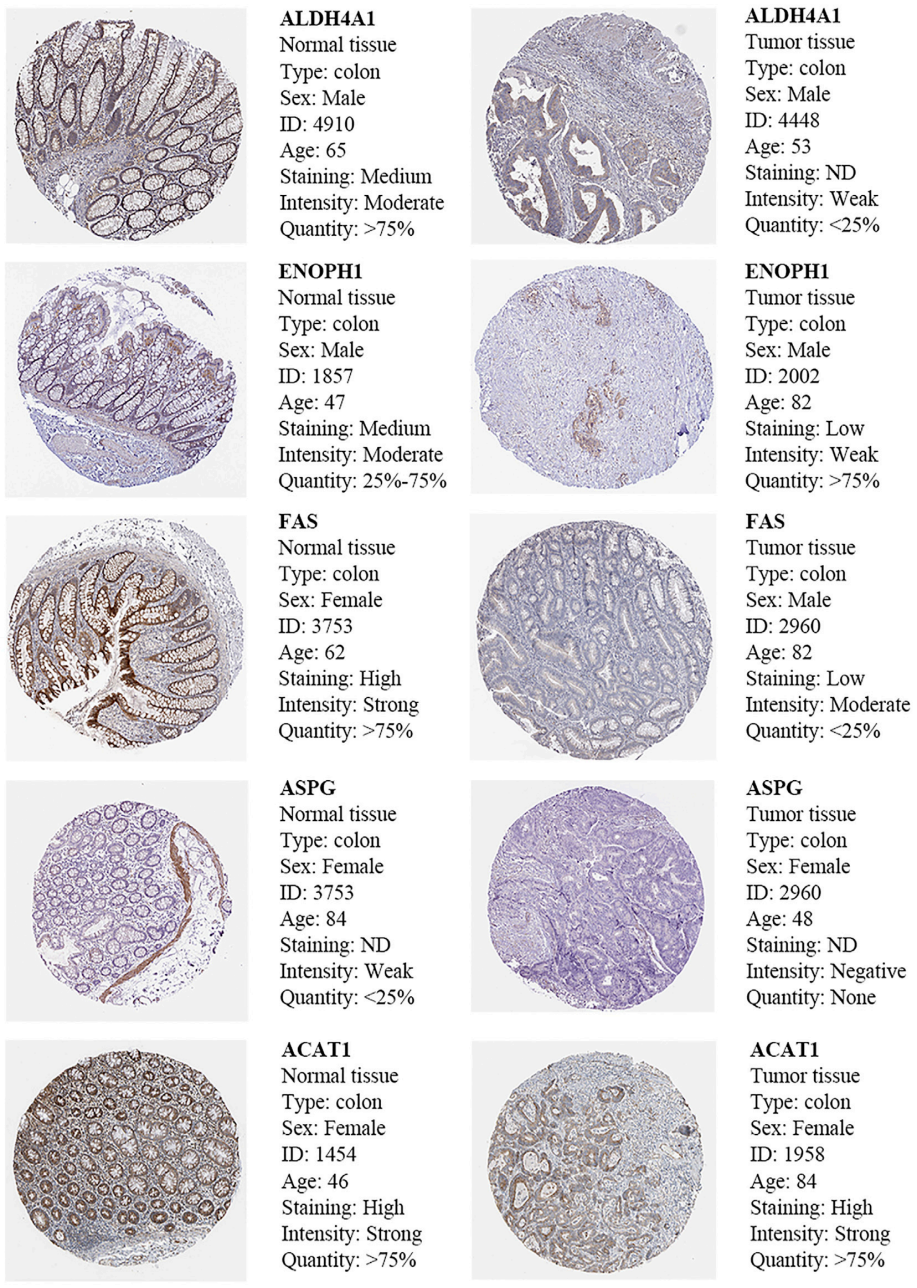
Immunohistochemistry and HE staining of five prognosis-related AAMRGs in the Human Protein Atlas database.

The results of immunohistochemistry (IHC) in the Human Protein Atlas (HPA) database showed that compared with normal tissues, ENOPH1, ACAT1, ALDH4A1, FAS, and ASPG are downregulated in cancer tissue than in normal tissue. These outcomes were consistent with our previous results (Figure 9).

## Discussion

CRC is a highly heterogeneous gastrointestinal tumor with high incidence and mortality worldwide (Sung et al., 2021). Due to the dissatisfaction with the results of targeted therapy and immunotherapy, it is necessary to develop alternative therapies. Since Otto Warburg discovered that tumor tissues utilized much more glucose than normal tissues in the 1920s, the altered metabolism of several amino acids in tumor tissues has also been gradually revealed (Hosios et al., 2016; Vettore et al., 2020). Furthermore, drugs targeting several amino acid metabolisms have been shown to inhibit the growth and invasion of colorectal cancer cells, for instance, the glutaminase inhibitor, CB-839, could enhance the antitumor activity of capecitabine and cetuximab (Cohen et al., 2020; Zhao et al., 2020).

We screened out five key genes to establish the risk signature based on the result of univariate cox and LASSO analyses, including ENOPH1, ACAT1, ALDH4A1, FAS, and ASPG. The survival analysis showed that the patients with high-risk scores had a worse prognosis, and the AUC of the risk signature was high. The risk score was an independent predictive factor for CRC no matter in univariate COX or multivariate COX analysis combined with clinical factors. In the nomogram integrating risk score with clinical factors, our risk score had the highest weighted score. The nomogram also had a high C-index and accuracy. Compared with earlier research (Ren’s 10-gene signature), our signature’s 5-years AUC was higher than Ren’s and was more concise with only five genes (Ren et al., 2022). These outcomes indicated our model was robust and performed well in predicting the survival of colorectal cancer patients.

The five genes in the model and their corresponding proteins all play important roles in amino acid metabolism. ACAT1, whose full name is acyl-coenzyme A cholesterol acyltransferase 1, is an enzyme involved in pathways of the tricarboxylic acid cycle, isoleucine degradation, ketogenic pathway, and ketolysis (Chen et al., 2019). The decrease of ACAT1 could inhibit the interaction among these metabolisms (Goudarzi, 2019; Min et al., 2020). Previous studies suggested that deficiency in ACAT1 activity mainly affected ketolysis (Korman, 2006; Sass, 2012; Goudarzi, 2019). The accumulation of beta-hydroxybutyrate, which is a more prevalent ketone body in CRC, could promote proliferation, invasion, and self-renewal potential of colorectal cancer cells (Shakery et al., 2018). Liu et al. (2022) discovered that the gene expression level of ACAT1 was obviously lower in CRC than in normal colorectal tissues, and decreased ACAT1 was strongly correlated to a worse prognosis of CRC, which is consistent with our result. The catalysis of aldehyde dehydrogenase 4A1 (ALDH4A1) is the final step of both proline and hydroxyproline catabolism (Bogner et al., 2021). Therefore, the deficiency of ALDH4A1 could lead to the accumulation of proline, which sustains the proliferation and survival of colorectal cancer cells (Namavar et al., 2021; Alaqbi et al., 2022). FAS, the death receptor for FASL of cytotoxic T lymphocytes, plays a significant role in suppressing the progression and metastasis of colorectal cancer (Gu et al., 2020; Merting et al., 2022). It was reported that the expression of FAS is dramatically downregulated in metastatic human colorectal cancer (Gu et al., 2020). ASPG, asparaginase, reduces the concentration of L-asparagine, L-glutamine, and glycine, three amino acids that are important components in the synthesis of purine and pyrimidine rings. The decrease of ASPG leads to the increase of asparagine, which supports tumor growth. A vivo research demonstrated that asparagine depletion could obviously inhibit the tumor growth of KRAS-mutant CRC cells (Hanada et al., 2021). Enolase-phosphatase 1 (ENOPH1), whose protein is an enzyme that participates in L-methionine biosynthesis, has been discovered to enhance the progression and invasion of glioma and hepatocellular carcinoma but has not been reported in colorectal cancer (Zhuang et al., 2019; Wang et al., 2021). The function of ACAT1, ALDH4A1, and FAS in colorectal cancer has been verified by previous studies, participating in the progression of CRC. However, there are no direct reports of ENOPH1 and ASPG on CRC, this study was the first to report that ENOPH1 and ASPG were associated with the prognosis of CRC. Our discovery might provide a new biomarker of CRC to be explored for follow-up research.

The results of drug sensitivity prediction showed that the patients with high-risk scores had higher sensitivity to dabrafenib, and the sensitivity of the two groups to commonly used antitumor drugs was similar. It was reported that dabrafenib and trametinib showed efficacy in patients with BRAF V600-mutant mCRC (Corcoran et al., 2015). The relationship between amino acid metabolism and the efficacy of dabrafenib requires further study.

Furthermore, we also conducted immune-related analysis on this model. The overall immune score of the patients with the low-risk scores was higher, indicating that immune infiltration was more abundant in the tumor microenvironment in the low-risk subgroup. The ssGSEA value of immune cells and the expression levels of immune checkpoints in the two subgroups showed that the activity of many immune cells and the expression levels of the majority of immune checkpoints in the low-risk samples were higher than those in the high-risk samples. The increase of immune checkpoints in the microenvironment leads to the immune cells differentiating into a state of inhibitory high expression, inhibiting anti-tumor immunity. Based on such immune cell infiltration and their activation status, the low-risk samples could be assigned to inflamed tumors (Nishikawa and Koyama, 2021). According to previous research, this kind of tumor immune microenvironment would have a stronger response to immunotherapy (Bai et al., 2021).

To verify this hypothesis, we obtained the TIDE score and TCIA immunophenoscore to predict the samples’ sensitivity to immunotherapy. The results showed the patients with the low-risk scores had lower TIDE scores; in other words, they were more positive toward immune therapy. Low-risk patients with double-positive CTLA4 and PD-1 and single-positive CTLA4 or PD-1 had higher immunophenoscores, which indicated that the low-risk group may respond stronger to anti-PD-1/PD-L1 or anti-CTLA-4 therapies. To sum up, this 5-AAMRG model might be useful in screening patients before immune checkpoint inhibitors (ICI) therapy. At present, PD-L1 tumor proportion score, TMB, mismatch repair deficiency, and microsatellite instability are being used to select patients who could benefit from ICI treatment (Yi et al., 2018). In our study, the predictive ability of this model is independent of mismatch repair deficiency and TMB, which means that our 5-gene signature may supplement the identification of potential beneficiaries under the current conditions.

The relationship between the risk signature and the immune landscape of CRC may be one of the main reasons for its good predictive ability. To be specific, the activity of a lot of tumor-infiltrating lymphocytes and myeloid cells in the low-risk samples was higher than that in the high-risk samples. The high infiltration and activation of CD8^+^ T cells are positively associated with the prognosis of CRC, suppressing metastasis development of colorectal cancer (Camus et al., 2009; Bruni et al., 2020). D4+T cell subsets had a complicated influence on tumor progression, including Th2, Th17, Tfh and so on (Galon and Bruni, 2020; Ben Khelil et al., 2022). Th17 cells could enhance anti-tumor immunity by producing IL-17 to induce the polarization of M1 macrophage and recruiting anti-tumor immune cells, for example, NK and CD8^+^ T cells (Al Omar et al., 2013; Guéry and Hugues, 2015; Jang et al., 2017). B cells, as the main effector cells of the adaptive immune response, are reported that increased B cell count is related to a better clinical prognosis of CRC (Edin et al., 2019). And Tfh cells were crucial for the maturation and activation of B cells (Bai et al., 2021). The interaction between Tfh cell and B cell contributes to the formation of anti-tumor immune structures (Galon et al., 2013). The role of the Th2 cell in CRC is controversial; it contributed to both antitumor and protumor responses by activating NK cells and inducing M2 macrophage polarization, respectively (De Monte et al., 2011; Lorvik et al., 2016). The functional deficiency of DC was associated with tumor-escape mechanism, metastasis initiation, and treatment resistance in CRC (Legitimo et al., 2014; Subtil et al., 2021). Accumulating evidence has shown that myeloid cells, including tumor-associated neutrophils, tumor-associated macrophages, eosinophils, mast cells, and MDSCs play an important role in coordinating cancer-associated immunosuppression and immune tolerance (Engblom et al., 2016; Mizuno et al., 2019; Kos et al., 2022). In addition, the amino acid metabolic reprogramming also could happen in the immune cells. Keshet et al. (2018) reported that the alteration of glutamine could promote the tumor-infiltrating T cells and impact tumor progression through other cells in the microenvironment. On the whole, the higher the activity of the normal amino acid metabolism pathway, the higher the likelihood of benefiting from immunotherapy, and the specific effects of the amino acid metabolism on the tumor immune environment were complex. Further research is needed to validate this result.

Through the comparison of clinical factors between the two risk subgroups, we discovered that a higher risk score means more lymph node metastasis and a more advanced stage. On the other hand, the heatmap of ssGSEA analysis showed that except for the processes of glutamine, histidine, lysine, tyrosine, and L-phenylalanine metabolism, the other amino acid metabolism pathways were less active in the high-risk subgroup. However, the synthesis and transportation of most amino acids did not change much. We suspected that as the tumor became more malignant and invasive, its amino acid metabolism pattern changed, and amino acids were synthesized and transported for abnormal metabolism pathways rather than normal amino acid metabolism pathways. The underlying metabolism of this phenomenon needs more research in the future.

Combing the results of ssGSEA and GSEA of hallmark pathways, Hedgehog signaling, WNT/β-catenin signaling, mitotic, myogenesis, Notch signaling, and TGF-β signaling were activated in the high-risk group. Epithelial–mesenchymal transition (EMT) is a key biological process for epithelial-derived tumor cells to acquire the ability of migration and invasion (Guarino et al., 2007). WNT/β-catenin signaling and TGF-β signaling pathway were vital pathways inducing EMT (Gu et al., 2019; Zhou et al., 2022). Abnormal activation of the Hedgehog signaling pathway can lead to the occurrence and progression of colorectal cancer (Guo et al., 2022). The enhancement of mitotic spindle and myogenesis pathways indicated that cell division, microfilament cytoskeleton, and stress fibers formation process were active (Caporali et al., 2021). The downregulation of pancreas beta cells, oxidative phosphorylation, peroxisome, and mTORC1 signaling showed the disturbance of nutrient metabolism of samples in the high-risk subgroup. These outcomes suggested that tumor cell proliferation and metastasis activity were more intense in the high-risk subgroup, and these pathways might be the key anti-tumor pathways for targeting amino acid metabolic therapy.

Our findings could provide novel perspectives for the formulation of individual precision medical programs. For instance, the signature can be applied as a supplement in identifying the potential beneficiaries of immunotherapy. Meanwhile, corresponding targeted drugs could be developed based on our new-discovered tumor-related biomarkers and changes in amino acid metabolic pathways, and the treatment could be carried out according to different features of amino acid metabolism in different CRC patients.

Undeniably, our study had some limitations. First, as the data in this research were downloaded from public databases, the AAMRG signature and its relationship with immune therapy response still need to be validated and revised by more retrospective and prospective studies. Second, part of the results of the amino acid metabolism in CRC samples has not been experimentally verified. In the future, we can use more bioinformatics tools such as the feature-level fusion (FLF) method (Jin and Sinicrope, 2022), explore the possibility of combining targeting amino acid metabolism therapy with copper-metal organic frameworks (Akhavan-Sigari et al., 2022), and conduct more laboratory and clinical studies to further explore the underlying impact of amino acid metabolism in tumor prognosis and immune therapy response of CRC.

## Conclusion

In summary, we established a risk signature comprising five AAMRGs (ENOPH1, ACAT1, ALDH4A1, FAS, and ASPG), and a higher score of this model was associated with worse survival in CRC. In addition, its prediction efficiency was well-validated. The immune-related analysis showed that AAMRGs were associated with the immune status of the tumor microenvironment; patients with low-risk scores were more positive toward immune therapy, which can be used as a predictor of efficacy. Our gene function enrichment analysis offered a new direction for the exploration of molecular mechanisms and targeting amino acid metabolism therapy for CRC.

## Data Availability

The original contributions presented in the study are included in the article/Supplementary Material; further inquiries can be directed to the corresponding authors.
