# Supplementary material for: Amino acid metabolism genes associated with immunotherapy responses and clinical prognosis of colorectal cancer
Source: Front Mol Biosci. 2022 Aug 5;9:955705. doi: 10.3389/fmolb.2022.955705 (PMC9388734; doi:10.3389/fmolb.2022.955705)
Supplement: Supplementary file 1 [file Table1.DOCX]

**TABLE S1|** Amino acid metabolism-related pathways from GO

| Pathways | GO pathway | Oganism |
| --- | --- | --- |
| GOBP_AMINO_ACID_ACTIVATION | GO:0043038 | Homo sapiens |
| GOBP_AMINO_ACID_HOMEOSTASIS | GO:0080144 | Homo sapiens |
| GOBP_AMINO_ACID_IMPORT | GO:0043090 | Homo sapiens |
| GOBP_AMINO_ACID_IMPORT_ACROSS_PLASMA_MEMBRANE | GO:0089718 | Homo sapiens |
| GOBP_AMINO_ACID_SALVAGE | GO:0043102 | Homo sapiens |
| GOBP_AMINO_ACID_TRANSMEMBRANE_TRANSPORT | GO:0003333 | Homo sapiens |
| GOBP_AMINO_ACID_TRANSPORT | GO:0006865 | Homo sapiens |
| GOBP_CELLULAR_AMINO_ACID_BIOSYNTHETIC_PROCESS | GO:0008652 | Homo sapiens |
| GOBP_CELLULAR_AMINO_ACID_CATABOLIC_PROCESS | GO:0009063 | Homo sapiens |
| GOBP_CELLULAR_AMINO_ACID_METABOLIC_PROCESS | GO:0006520 | Homo sapiens |
| GOBP_REGULATION_OF_CELLULAR_AMINO_ACID_METABOLIC_PROCESS | GO:0006521 | Homo sapiens |
| GOBP_POSITIVE_REGULATION_OF_AMINO_ACID_TRANSPORT | GO:0051957 | Homo sapiens |
| GOBP_REGULATION_OF_AMINO_ACID_TRANSMEMBRANE_TRANSPORT | GO:1903789 | Homo sapiens |
| GOBP_REGULATION_OF_AMINO_ACID_TRANSPORT | GO:0051955 | Homo sapiens |
| GOBP_NEGATIVE_REGULATION_OF_AMINO_ACID_TRANSPORT | GO:0051956 | Homo sapiens |
| GOBP_RESPONSE_TO_AMINO_ACID_STARVATION | GO:1990928 | Homo sapiens |

**TABLE S2|** Amino acid metabolism-related genes

| Pathways | Genes |
| --- | --- |
| GOBP_AMINO_ACID_ACTIVATION | FARSB, WARS2, FARS2, TARS3, EARS2, AASDH, AARS1, DARS1, EPRS1, FARSA, LARS2, HARS2, PARS2, GARS1, GATC, HARS1, IARS1, KARS1, MARS1, NARS1, YARS2, LARS1, GATB, SARS2, DALRD3, DARS2, QRSL1, IARS2, RARS2, VARS2, LRRC47, AARS2, QARS1, RARS1, SARS1, TARS1, VARS1, WARS1, CARS2, NARS2, TARS2, AARSD1, CARS1, YARS1, MARS2 |
| GOBP_AMINO_ACID_HOMEOSTASIS | KCTD7, SLC7A11, GLS, GRM2, SLC66A1, SLC1A1, TPP2 |
| GOBP_AMINO_ACID_IMPORT | ARL6IP5, SLC6A14, SFXN2, SLC36A4, SLC43A2, AGT, CLN8, SLC36A1, TSPO2, ARL6IP1, SLC7A8, SLC7A11, SH3BP4, GFAP, GRM1, ITGB1, KCNJ10, ATP1A2, NTSR1, SLC6A20, SLC25A38, SLC47A1, PSEN1, CLTRN, ACE2, RGS2, RGS4, SLC1A1, SLC1A2, SLC1A3, SLC1A4, SLC1A5, SLC1A6, SLC3A2, SLC6A1, SLC6A6, SLC6A9, SLC6A11, SLC6A12, SLC6A13, SLC7A1, SLC7A2, SLC16A2, SLC22A2, SLC22A4, TNF, SLC7A5, SFXN3, SLC7A3, SLC43A1, PER2, SLC6A5, SLC9A3R1, SFXN1 |
| GOBP_AMINO_ACID_IMPORT_ACROSS_PLASMA_MEMBRANE | ARL6IP5, SLC6A14, SLC36A4, SLC43A2, AGT, CLN8, SLC36A1, TSPO2, ARL6IP1, SLC7A8, SLC7A11, GFAP, GRM1, ITGB1, KCNJ10, ATP1A2, NTSR1, SLC6A20, SLC47A1, PSEN1, CLTRN, ACE2, RGS2, RGS4, SLC1A1, SLC1A2, SLC1A3, SLC1A4, SLC1A5, SLC1A6, SLC3A2, SLC6A6, SLC6A9, SLC6A13, SLC7A1, SLC7A2, SLC16A2, SLC22A2, SLC22A4, TNF, SLC7A5, SLC7A3, SLC43A1, PER2, SLC6A5 |
| GOBP_AMINO_ACID_SALVAGE | BHMT2, MTAP, APIP, ADI1, ENOPH1, BHMT, MRI1 |
| GOBP_AMINO_ACID_TRANSMEMBRANE_TRANSPORT | SLC25A13, SLC25A15, ARL6IP5, SLC38A3, SLC7A9, PRAF2, SLC6A14, SLC16A10, SFXN2, CLN3, SLC36A4, SLC15A4, SLC25A29, SLC38A10, SLC43A2, MFSD12, SLC32A1, SLC38A6, SLC38A8, SLC38A11, SLC66A1L, SLC38A9, SLC36A2, SLC7A13, AGT, CLN8, SLC36A1, TSPO2, ARL6IP1, SLC7A8, SLC7A11, SLC17A8, GFAP, SLC36A3, GRM1, SLC6A19, SLC6A18, ITGB1, KCNJ10, SLC7A5P2, ATP1A2, NTSR1, SLC38A2, SLC6A20, SLC66A1, SLC25A38, SLC38A4, SLC6A15, LRRC8D, SLC38A7, SLC47A1, LRRC8A, SLC7A10, PSEN1, SLC17A7, SLC17A6, CLTRN, SLC7A14, ACE2, RGS2, RGS4, SLC1A1, SLC1A2, SLC1A3, SLC1A4, SLC1A5, SLC1A6, SLC1A7, SLC3A1, SLC3A2, SLC6A6, SLC6A7, SLC6A9, SLC6A12, SLC6A13, SLC7A1, SLC7A2, SLC7A4, SLC16A2, SLC22A2, SLC22A4, TNF, SLC25A22, LRRC8E, SLC7A5, SLC38A1, SFXN3, SLC7A5P1, SLC25A18, SLC25A2, LRRC8C, SLC7A3, SLC43A1, SLC25A12, PER2, SLC7A7, SLC7A6, SLC6A5, SLC38A5, SFXN1 |
| GOBP_AMINO_ACID_TRANSPORT | SLC25A13, SLC25A15, ARL6IP5, PDPN, GIPC1, SERINC3, SLC38A3, SLC7A9, PRAF2, SLC6A14, HRH3, SLC16A10, SFXN2, SFXN4, CLN3, SLC36A4, SLC15A4, SLC25A29, SLC38A10, SLC43A2, MFSD12, ADORA1, ADORA2A, SLC32A1, SLC38A6, SLC38A8, CTNS, SLC38A11, SLC66A1L, SLC38A9, SLC36A2, SLC25A48, SLC7A13, ABAT, AGT, CLN8, SLC36A1, TSPO2, RAB3GAP1, ARL6IP1, SLC7A8, SLC7A11, SH3BP4, SLC17A8, GABBR1, SERINC5, SLC17A5, GFAP, GJA1, SLC25A45, SLC25A47, SLC36A3, GRM1, GRM2, GRM7, APBA1, HTR1B, SLC6A19, SLC6A18, ITGB1, KCNJ10, SLC7A5P2, SLC6A17, LEP, LLGL2, MYC, NF1, ATP1A2, NPY5R, NTRK2, NTSR1, OCA2, P2RX7, SLC38A2, SLC6A20, SLC66A1, SLC25A38, SLC38A4, AVP, SLC6A15, LRRC8D, AVPR1A, SLC38A7, SLC47A1, AVPR1B, LRRC8A, SLC7A10, PSEN1, SLC17A7, SLC17A6, CLTRN, SLC7A14, ACE2, RGS2, RGS4, SLC1A1, SLC1A2, SLC1A3, SLC1A4, SLC1A5, SLC1A6, SLC1A7, SLC3A1, SLC3A2, SLC6A1, SLC6A6, SLC6A7, SLC6A9, SLC6A11, SLC6A12, SLC6A13, SLC7A1, SLC7A2, SLC7A4, SLC12A2, SLC16A2, SLC22A2, SLC22A4, SNCA, STXBP1, SYT4, TNF, TRH, TRPC4, TRPV1, XK, SLC25A22, LRRC8E, SLC7A5, SLC38A1, SFXN3, SLC7A5P1, SLC25A18, SLC25A2, DTNBP1, LRRC8C, SLC7A3, SLC43A1, KMO, SLC25A12""PER2, SLC7A7, SLC7A6, SLC6A5, SLC38A5, SLC9A3R1, SFXN1, SFXN5, SLC25A44, SV2A |
| GOBP_CELLULAR_AMINO_ACID_BIOSYNTHETIC_PROCESS | AASS, CBSL, SERINC3, SDS, ILVBL, PARK7, SDSL, CLN3, NOXRED1, CPS1, GOT1L1, CTH, NAGS, DHFR, ABAT, DPYD, AGXT, DHFR2, ALDH1A1, SEPHS2, SEPHS1, BHMT2, SERINC5, PHGDH, GGT1, GLS2, GLS, GLUD1, GLUD2, GLUL, GOT1, GOT2, PYCR2, PSAT1, ASL, ASNS, MTHFD2L, ASS1, MTAP, MTHFD1, MTHFR, MTR, MTRR, ATP2B4, OAT, OTC, PAH, PCBD1, APIP, LGSN, UPB1, PLOD2, ASNSD1, ADI1, PSPH, DHFRP1, PYCR1, ALDH18A1, ENOPH1, BCAT1, BCAT2, AASDHPPT, BHMT, SRR, SHMT1, SHMT2, AGXT2, SLC1A3, PYCR3, CAD, PCBD2, MRI1, SLC25A12, CBS, PSPHP1, PLOD3 |
| GOBP_CELLULAR_AMINO_ACID_CATABOLIC_PROCESS | AASS, RIDA, CBSL, BCKDK, CDO1, FTCD, SDS, HIBADH, HOGA1, SDSL, HYKK, AFMID, ACMSD, UROC1, ADHFE1, CARNMT1, CRYM, AMDHD1, TDH, DAO, DBT, IDO2, DLD, DLST, SARDH, ABAT, AGXT, ECHS1, ETFA, ETFB, FAH, GCAT, HAAO, DDAH2, DDAH1, GAD1, GAD2, IL4I1, HIBCH, GCSH, ACAD8, GLS2, GLDC, GLS, GLUD1, GLUD2, AMT, GLUL, GOT1, GOT2, GPT, GSTZ1, HSD17B10, HAL, HDC, HGD, HMGCL, HNMT, HPD, ACADSB, IDO1, IVD, ACAT1, ARG1, ARG2, MIR21, MAT1A, ALDH6A1, ASPA, MTRR, NOS1, NOS2, NOS3, ATP2B4, OAT, OTC, PAH, AADAT, PIPOX, CSAD, HMGCLL1, AUH, ENOSF1, PRODH, KYAT3, MCCC1, CARNS1, PRODH2, QDPR, BCAT2, BCKDHA, BCKDHB, MCCC2, BLMH, ALDH8A1, SHMT1, SHMT2, AGXT2, TAT, TDO2, ALDH5A1, ASRGL1, GPT2, DDO, KMO, ALDH4A1, CBS, KYAT1, KYNU, ARHGAP11B, SLC25A21, SLC25A44 |
| GOBP_CELLULAR_AMINO_ACID_METABOLIC_PROCESS | GLYATL1B, FARSB, AASS, SLC25A13, RIDA, GLYAT, CBSL, BCKDK, WARS2, CDO1, MTHFS, FARS2, FTCD, SERINC3, SDS, ILVBL, HIBADH, HOGA1, PARK7, AZIN2, SDSL, CLN3, TPH2, NOXRED1, TARS3, HYKK, EARS2, AFMID, ACMSD, UROC1, AASDH, CPS1, GOT1L1, ADHFE1, CARNMT1, CRYM, TTC36, AMDHD1, PM20D1, CTH, CTNS, CTPS1, TDH, ADSS2, AARS1, DAO, DARS1, NAGS, DBT, DCT, DDC, IDO2, DHFR, DIO1, DLD, DLST, SARDH, ABAT, DPEP1, DPYD, AGXT, ECHS1, DHFR2, EPRS1, ETFA, ETFB, ALDH1A1, FAH, FARSA, SEPHS2, SEPHS1, LARS2, SIRT4, HARS2, ICMT, GCAT, HAAO, FPGS, DDAH2, DDAH1, SLC7A11, BHMT2, SERINC5, GAD1, GAD2, IL4I1, PARS2, GARS1, GART, PHGDH, BLOC1S6, HIBCH, GCDH, GCSH, GFPT1, GGT1, GGT5, ACAD8, GLS2, GNMT, GCLC, GCLM, GLDC, GLS, GLUD1, GLUD2, AMT, GLUL, GOT1, GOT2, GATC, RIMKLA, GPT, GSS, GSTZ1, PYCR2, PSAT1, HSD17B10, HAL, HARS1, HDC, HGD, HMGCL, HNF4A, HNMT, HPD, IARS1, NAT8L, ACADSB, IDO1, INS, IVD, KARS1, ASPG, ACAT1, ARG1, ARG2, IYD, ACCSL, MIR21, MARS1, MAT1A, ART4, MECP2, ALDH6A1, ASL, MPST, ASNS, MTHFD2L, ASPA, ASS1, MSRA, MTAP, MTHFD1, MTHFR, MTR, MTRR, MMUT, NARS1, ATF4, NOS1, NOS2, NOS3, ATP2B4, OAT, ODC1, OTC, NOX4, PAH, PCBD1, YARS2, APIP, THAP4, AADAT, PIPOX, CSAD, LARS1, SCLY, LGSN, AZIN1, UPB1, PEPD, GATB, PFAS, PLOD2, ATP7A, HMGCLL1, ASNSD1, PPAT, AUH, SARS2, DALRD3, DARS2, ADI1, THNSL2, QRSL1, ENOSF1, IARS2, PRODH, KYAT3, CTPS2, MCCC1, NIT2, BAAT, RARS2, VARS2, PSPH, DHFRP1, LRRC47, RIMKLB, AARS2, CARNS1, PTS, PYCR1, ALDH18A1, ENOPH1, PRODH2, QARS1, BCAT1, QDPR, BCAT2, RARS1, BCKDHA, BCKDHB, AASDHPPT, SARS1, BHMT, SRR, MCCC2, SLC39A8, FN3K, BLMH, ALDH8A1, SHMT1, SHMT2, AGXT2, SLC1A3, PYCR3, SLC16A2, SMS, BPHL, TARS1, TAT, TDO2, TH, TPH1, TST, TYR, VARS1, WARS1, CAD, ALDH5A1, CARS2, NARS2, AGMAT, DGLUCY, ASRGL1, TARS2, AARSD1, CARS1, PCBD2, MRI1, ACCS, GPT2, HPDL, DDO, ATCAY, KMO, YARS1, SLC25A12, ALDH4A1, CBS, PSPHP1, KYAT1, GMPS, KYNU, ARHGAP11B, PLOD3, SLC25A21, SLC7A7, GLYATL1, MARS2, ACY1, SLC25A44, GFPT2, NR1H4 |
| GOBP_REGULATION_OF_CELLULAR_AMINO_ACID_METABOLIC_PROCESS | BCKDK, PARK7, CLN3, ACMSD, SIRT4, SLC7A11, INS, MIR21, ATP2B4, BHMT, ATCAY, SLC7A7, NR1H4 |
| GOBP_POSITIVE_REGULATION_OF_AMINO_ACID_TRANSPORT | SLC38A3, ADORA2A, ABAT, AGT, RAB3GAP1, ARL6IP1, GABBR1, ITGB1, NTSR1, P2RX7, AVP, AVPR1A, AVPR1B, PSEN1, CLTRN, ACE2, SLC6A1, SLC12A2, STXBP1, SYT4, TRH, DTNBP1, KMO |
| GOBP_REGULATION_OF_AMINO_ACID_TRANSMEMBRANE_TRANSPORT | ARL6IP5, SLC43A2, AGT, ARL6IP1, ITGB1, ATP1A2, PSEN1, CLTRN, ACE2, RGS2, RGS4, TNF, SLC43A1, PER2 |
| GOBP_REGULATION_OF_AMINO_ACID_TRANSPORT | ARL6IP5, SLC38A3, HRH3, SLC43A2, ADORA1, ADORA2A, ABAT, AGT, RAB3GAP1, ARL6IP1, GABBR1, GRM2, GRM7, HTR1B, ITGB1, LEP, ATP1A2, NPY5R, NTSR1, P2RX7, AVP, AVPR1A, AVPR1B, PSEN1, CLTRN, ACE2, RGS2, RGS4, SLC6A1, SLC12A2, SNCA, STXBP1, SYT4, TNF, TRH, DTNBP1, SLC43A1, KMO, PER2, SV2A |
| GOBP_NEGATIVE_REGULATION_OF_AMINO_ACID_TRANSPORT | ARL6IP5, HRH3, SLC43A2, ADORA1, ABAT, GABBR1, GRM7, HTR1B, LEP, NPY5R, RGS2, RGS4, TNF, TRH, SLC43A1 |
| GOBP_RESPONSE_TO_AMINO_ACID_STARVATION | CDKN1A, RRAGB, NPRL2, RRAGA, GCN1, KPTN, ATF2, SESN3, KICS2, BMT2, DAP, EIF2S1, FLCN, RNF152, SZT2, LARP1, MTOR, SESN1, FAS, MAP3K5, EIF2AK4, ATF3, ATF4, SH3GLB1, LARS1, SLC38A2, MIOS, IMPACT, ITFG2, PRKD1, MAPK1, MAPK3, MAPK8, EIF2AK2, RRAGD, RRAGC, UCP2, TFEB, WDR59, NPRL3, SEH1L, SESN2, EIF2A, WDR24, MAP1LC3A, BECN1, DAPL1, EIF2AK3, DEPDC5 |

**TABLE S3|** Amino acid biosynthetic and transport pathways from GO

| Pathways | Oganism |
| --- | --- |
| GOBP_ASPARTATE_FAMILY_AMINO_ACID_BIOSYNTHETIC_PROCESS | Homo sapiens |
| GOBP_GLUTAMATE_BIOSYNTHETIC_PROCESS | Homo sapiens |
| GOBP_L_PROLINE_BIOSYNTHETIC_PROCESS | Homo sapiens |
| GOBP_L_SERINE_BIOSYNTHETIC_PROCESS | Homo sapiens |
| GOBP_ALANINE_TRANSPORT | Homo sapiens |
| GOBP_GLUTAMINE_TRANSPORT | Homo sapiens |
| GOBP_L_ALANINE_TRANSPORT | Homo sapiens |
| GOBP_L_GLUTAMATE_TRANSMEMBRANE_TRANSPORT | Homo sapiens |
| GOBP_L_HISTIDINE_TRANSMEMBRANE_TRANSPORT | Homo sapiens |
| GOBP_L_LYSINE_TRANSMEMBRANE_TRANSPORT | Homo sapiens |
| GOBP_L_SERINE_TRANSPORT | Homo sapiens |
| GOBP_LEUCINE_TRANSPORT | Homo sapiens |
| GOBP_PROLINE_TRANSMEMBRANE_TRANSPORT | Homo sapiens |
| GOBP_PROLINE_TRANSPORT | Homo sapiens |
| GOBP_SERINE_TRANSPORT | Homo sapiens |
| GOBP_SULFUR_AMINO_ACID_TRANSPORT | Homo sapiens |
| GOBP_TRYPTOPHAN_TRANSPORT | Homo sapiens |
